# Emphysematous gastritis: A case series of three patients managed conservatively

**DOI:** 10.1016/j.ijscr.2019.09.046

**Published:** 2019-10-07

**Authors:** Hassan Nasser, Tommy Ivanics, Shravan Leonard-Murali, Dania Shakaroun, Ann Woodward

**Affiliations:** a Department of Surgery, Henry Ford Hospital, 2799 W Grand Blvd, Detroit, MI, 48202, USA; b Department of Internal Medicine, Henry Ford Hospital, 2799 W Grand Blvd, Detroit, MI, 48202, USA

**Keywords:** Case series, Emphysematous gastritis, Gastric emphysema, Gastritis, Computed tomography

## Abstract

**Introduction:**

Emphysematous gastritis (EG) is a rare condition characterized by air within the gastric wall with signs of systemic toxicity. The optimal management for this condition and the role of surgery is still unclear. We here report three cases of EG successfully managed non-operatively.

**Presentation of cases:**

All three of our patients were elderly females with several **co-**morbidities. The chief presenting symptom was abdominal pain with signs of systemic toxicity ranging from tachycardia and hypotension to acute kidney injury. Computed tomography (CT) scan revealed gastric pneumatosis in all patients. One patient had extensive portal venous gas, and another had free intraperitoneal air. All patients were managed with nothing by mouth, proton pump inhibitors, intravenous fluid resuscitation, and antibiotics. Repeat CT scan in two patients in 3–4 days demonstrated resolution of the pneumatosis. They were all discharged home tolerating an oral diet.

**Discussion:**

The presentation of EG is non-specific and the diagnosis is primarily established by findings of intramural air in the stomach on CT scan. The initial management of EG should be nothing by mouth, proton pump inhibitor, intravenous fluid resuscitation, and antibiotics with surgical exploration only reserved for cases that fail non-operative management, demonstrate clinical deterioration, or develop signs of peritonitis.

**Conclusion:**

Early recognition and initiation of appropriate therapy is crucial to prevent the progression of EG. EG, even in the presence of portal venous air or pneumoperitoneum, should not represent a sole indication for surgical exploration and trial of initial non-operative management should be attempted when clinically appropriate.

## Highlights

• Computed tomography scan is the best test to establish the diagnosis of EG.
• Early recognition and initiation of therapy is crucial to prevent progression of EG.
• Surgical exploration is indicated after failure of non-operative management.

## 1 Introduction

Emphysematous gastritis (EG) is a rare infection of the stomach wall by invasive gas-forming organisms. Gram-positive, gram-negative, anaerobic, and fungal organisms have been implicated in the pathogenesis of EG and commonly isolated organisms include *Streptococcus* species, *Escherichia coli*, *Enterobacter* species, *Clostridium* species, *Staphylococcus aureus*, *Klebsiella pneumoniae*, *Pseudomonas aeruginosa*, and *Candida* species [1,2]. In 42.4% of cases however, no organism is identified [3]. In a recently published review, Watson et al. reported 59 cases of EG up to June 2014 in the English literature with a reported mortality rate of 47.5% [3]. Predisposing factors for EG include malignancy, caustic ingestion, recent surgery, bowel obstruction, gastric distension, emesis, steroids, immunosuppressive medications, chemotherapy, alcohol, and nonsteroidal ant-inflammatory drugs [[1], [2], [3], [4]]. EG typically presents with abdominal pain, nausea, vomiting, diarrhea, and occasionally hematemesis and sepsis [5,6]. Computed tomography (CT) scan is the most effective diagnostic modality to detect intramural emphysema [7]. Several studies emphasize the distinction between EG and gastric emphysema (GE). GE is reported to be a relatively benign condition where gas is seen in the wall of the stomach and is thought to occur secondary to barotrauma with no associated signs of infection or systemic toxicity [4]. Nevertheless, because of the overlap in the presentation between EG and GE, distinguishing between these conditions may be challenging [1,5] Over the last two decades, the role of surgical exploration in patients with EG has been questioned and the optimal treatment strategy remains unclear [3]. We report three cases of EG which were managed non-operatively without any mortality. This case series has been reported in line with the PROCESS guidelines [8].

## 2 Presentation of cases

This is a retrospective case series which involves three patients who presented to our academic institution between March 2018 and June 2018 with emphysematous gastritis. This study was exempt from ethical approval at our institution.

### 2.1 Case 1

**Fig. 1:**
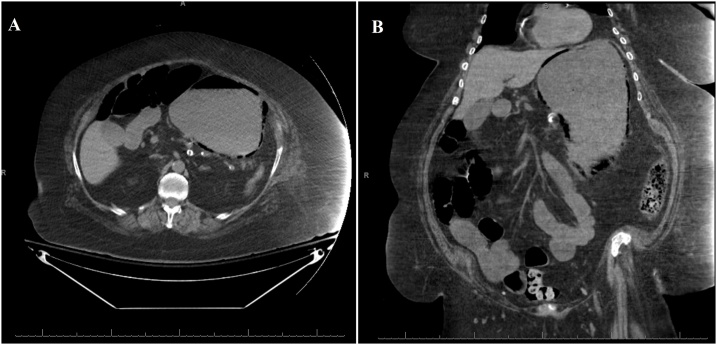
Non-contrast computed tomography scan of the abdomen revealed gastric distention and gastric pneumatosis throughout the wall of the stomach.

**Fig. 2:**
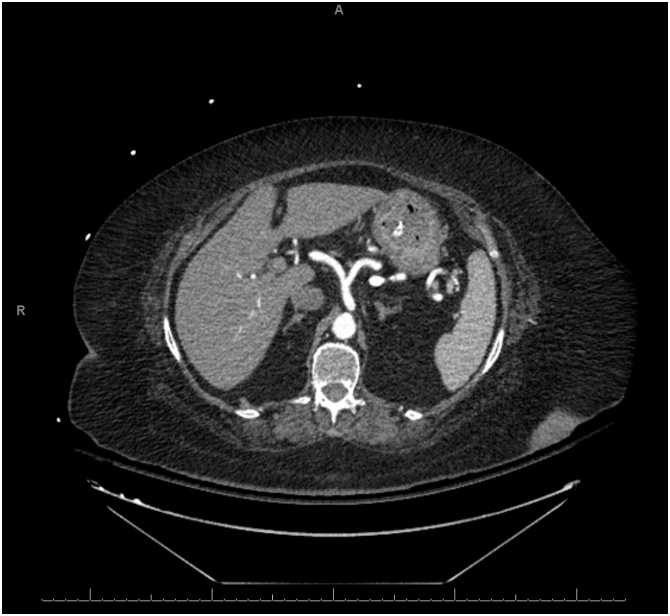
Computed tomography scan of the abdomen showing resolution of the gastric pneumatosis after 4 days.

A 78-year-old female presented to an outside hospital with one-week history of diffuse abdominal pain with associated nausea, vomiting, and diarrhea. Her past medical history was significant for factor V Leiden, deep vein thrombosis, pulmonary embolism, hypertension, diabetes mellitus, morbid obesity, atrial fibrillation, heart failure with preserved ejection fraction, and chronic obstructive pulmonary disease on 2 liters home oxygen. She was on warfarin and had an inferior vena cava filter in place for her history of venous thromboembolic disease and thrombophilia. Her vital signs were significant for tachycardia. Abdominal examination was soft, non-distended, minimally tender to palpation in the epigastrium without rebound or guarding. Initial laboratory studies were significant for a **white** blood cell count (WBC) of 21,600 cells/μL, serum creatinine of 2.18 mg/dL, lactate of 2.7 mmol/L, and international normalized ratio (INR) of 5.36. Her INR was corrected with vitamin K and fresh frozen plasma and the patient was transitioned to an intravenous unfractionated heparin infusion. CT scan of the abdomen without contrast was obtained to evaluate her abdominal pain and revealed gastric distension with gastric pneumatosis (Fig. 1). She was treated for EG with nasogastric tube (NGT) decompression, intravenous fluid resuscitation, nil per os (NPO), antibiotics (vancomycin, cefepime, metronidazole, and fluconazole), and proton pump inhibitor (PPI). Two days after admission, she developed bloody output through her NGT and dropped her hemoglobin from 11.2 to 7.8 g/dL. Her heparin infusion was stopped. At this point, she was transferred to our medical intensive care unit for escalation of care. On arrival the patient was hemodynamically stable with coffee-ground output from her NGT. She reported that her abdominal pain had improved significantly. Laboratory studies eventually revealed a normalization of her WBC count, serum creatinine, and lactic acid. Her hemoglobin stabilized without further need for transfusion. Decision was made to not pursue an esophagogastroduodenoscopy (EGD) given no further evidence of bleeding and improvement in her pain. General surgery was consulted and recommended non-operative management. After four days from the first CT scan, a CT angiography of the abdomen with contrast was obtained to evaluate for vascular patency which revealed patent celiac trunk and resolution of her gastric pneumatosis (Fig. 2). The NGT was then removed and the patient tolerated an oral diet. She completed a total of seven days of antibiotics. She was discharged home on PPI therapy and warfarin was restarted.

### 2.2 Case 2

**Fig. 3:**
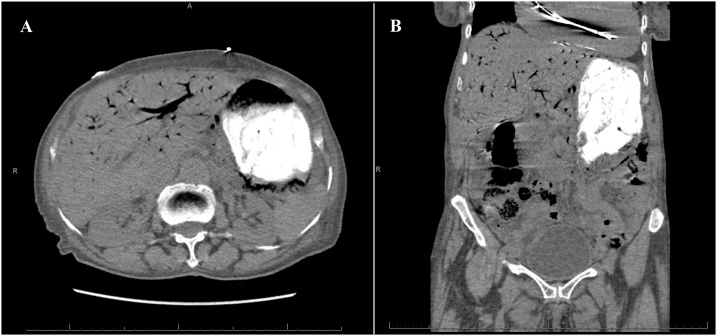
Non-contrast computed tomography scan on presentation revealing gastric pneumotosis and extensive portal venous gas.

**Fig. 4:**
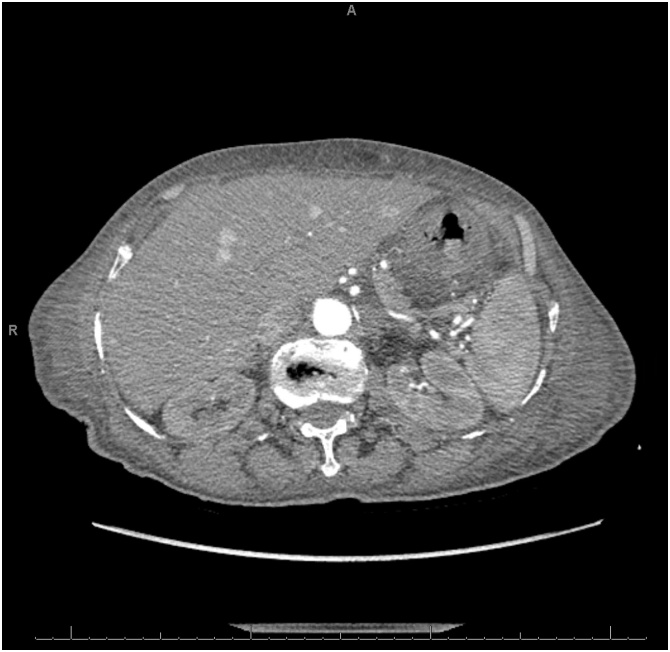
Computed tomography scan of the abdomen with contrast three days after the first scan showing resolution of the gastric pneumatosis and portal venous gas.

An 87-year-old presented to our emergency department with four days history of diffuse abdominal pain and associated non-bilious non-bloody emesis. She also complained of worsening shortness of breath and bilateral lower extremity edema over the last two weeks. She has a past medical history significant for congestive heart failure (CHF) with ejection fraction of 22%, pulmonary hypertension, and atrial fibrillation. She was on carvedilol and bumetanide. However, she has been non-compliant with her diuretic therapy. Her vital signs were significant for hypotension of 90/40. Abdominal exam was soft, non-distended, and non-tender without signs of peritonitis. Lung exam revealed clear lungs bilaterally without wheezing or crackles. She had bilateral lower extremity edema on exam. Laboratory studies showed a normal WBC of 7.0 cells/μL, creatinine of 1.04 mg/dL (baseline of 0.75 mg/dL), brain natriuretic peptide (BNP) of 868 pg/mL (reference <50 pg/mL), and lactate of 1.9 mmol/L. Electrocardiogram and serum troponin level were unremarkable. Chest roentgenogram revealed cardiomegaly with no evidence of pulmonary edema. CT scan of the abdomen without contrast revealed gastric pneumatosis with portal venous gas throughout the liver (Fig. 3). The patient was admitted and restarted on her home bumetanide for possible CHF exacerbation. Her EG was managed non-operatively with PPI therapy, antibiotics (ceftriaxone and metronidazole), and NPO given her benign exam and hemodynamic stability. An EGD was not performed. Three days after admission, a repeat CT scan of the abdomen with contrast was obtained which showed resolution of the gastric pneumatosis and portal venous gas (Fig. 4). Her pain has resolved at this time and she was started on an oral diet. After completing a total of seven days of antibiotics, she was discharged home on PPI therapy tolerating oral intake. The patient continues to do well two months after discharge.

### 2.3 Case 3

**Fig. 5:**
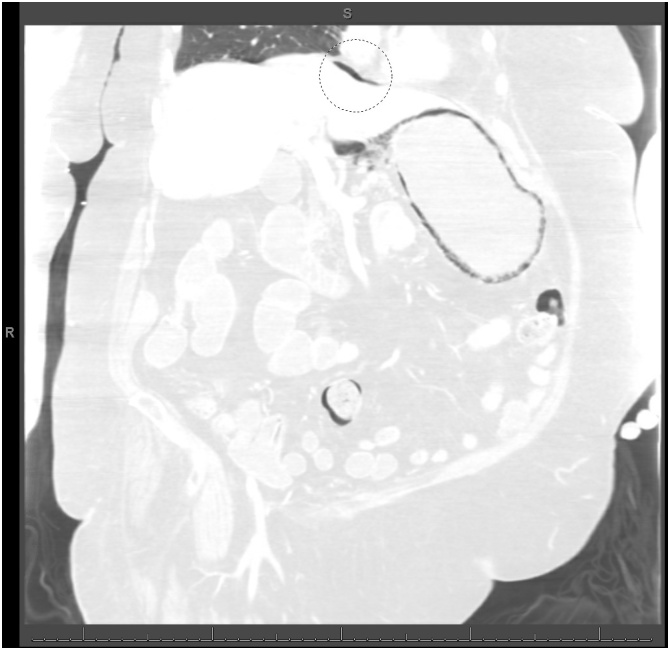
Computed tomography scan of the abdomen (lung window) showing extensive gastric pneumatosis and free intraperitoneal air superior to the liver (circle).

A 78-year-old female was brought into the emergency department from a long-term acute care facility for a chief complaint of mild diffuse abdominal pain over the last two days. She reported associated nausea and vomiting. Her past medical and surgical history were significant for bipolar disorder, congestive heart failure, hypertension, hypothyroidism, gastroesophageal reflux disease, and status post appendectomy. On arrival, her vital signs were significant for hypotension of 95/41. Her abdominal exam was soft, non-tender, and non-distended without signs of peritonitis. Her laboratory studies showed leukocytosis of 21,100 cells/μL and serum creatinine of 1.88 mg/dL (baseline of 0.7 mg/dL). CT scan of the abdomen with contrast revealed gastric pneumatosis with free intraperitoneal air (Fig. 5). The patient was treated non-operatively with NGT decompression, NPO, intravenous fluid resuscitation, PPI, and antibiotics (piperacillin/tazobactam). An EGD was not performed. After three days, her pain gradually improved and her WBC and serum creatinine normalized. The NGT was removed and she was started on an oral diet. She was discharged back to her long-term acute care facility after completing a 1-week course piperacillin/tazobactam. The patient was admitted to another hospital two months later and expired from multi-organ failure from an unclear etiology.

## 3 Discussion

EG is a rare but potentially fatal condition which is characterized by the presence of air in the wall of the stomach with associated systemic toxicity. Clinical presentation of EG is relatively non-specific and is typically characterized by abdominal pain, nausea, vomiting, and occasionally hematemesis. Physical examination findings vary depending on the degree of severity but can range from mild abdominal tenderness to peritonitis, especially in the setting of perforation. A distinction between EG and GE has been described in the literature. Since the two conditions have identical radiographic findings of gastric pneumatosis with an almost equal association with portal venous gas and pneumoperitoneum [1], we believe that the two entities likely represent various severities on the spectrum of the same disease. Distinction is primarily based on the severity of systemic toxicity and hemodynamic instability. Although the exact pathophysiology behind EG is not clearly understood, an ischemic injury to the gastric wall seems to be the inciting event for EG. This ischemic injury may lead to a secondary infection either from local bacterial invasion through the ulceration or from hematogenous spread [3,9]. In the cases we reported, only the second patient had a clear inciting event which was most likely an acute CHF exacerbation with a low flow state. The other two patients lacked a clear inciting event.

The diagnosis of EG is most commonly and best established on CT scan of the abdomen, although abdominal roentgenogram may be sufficient to make the diagnosis [3,7]. The extent of gastric emphysema as well as presence of portal venous gas and pneumoperitoneum in this setting do not correlate with the severity of disease or need for operative management [4,10,11]. Two of our reported cases had portal venous gas or pneumoperitoneum but did not require exploration given their relative stability, absence of peritoneal signs, and response to conservative management. An interesting finding that was noted in two of our cases that had a repeat CT scan in the first 3–4 days after diagnosis is the rapid resolution of gastric pneumatosis which coincided with improvement in symptoms. EGD usually identifies an inflamed, erosive, or necrotic area of mucosa in patient with EG [9]. The role of EGD in the diagnosis of EG has not been clearly defined despite its increased use in the management of EG in the last two decades [3]. Matsushima et al. recommended an EGD as part of the algorithm in the management of EG and the presence of ischemic gastric mucosa as an indication for surgical exploration [1]. However, Robinson et al. reported a case of EG with evidence of necrotic mucosa on EGD and portal venous gas on CT scan where non-operative management was chosen given hemodynamic stability with good outcome [4]. Alvin et al. described a case of a patient who underwent surgical exploration due to findings of severe erosive and necrotic gastritis on EGD without noting any evidence of gastric ischemia on subsequent exploration [12]. Thus, we believe that EGD findings are poor predictors for the presence of transmural ischemia and that operating solely on EGD findings of necrosis may lead to unnecessary extensive surgical interventions. On the other hand, EGD may have a role in patients who have clinical deterioration and surgical exploration is being considered [13]. EGD allows identification of the offending organism by culturing mucosal samples and allowing tailoring of antibiotic therapy accordingly. None of our patients underwent an EGD given clinical improvement with conservative management.

The management of EG initially includes intravenous fluid resuscitation, NPO, PPI, and broad-spectrum antibiotics covering gram negative and anaerobic organisms. The duration of antibiotic therapy is not well established, and we chose to only treat with 7 days as all three of our patients improved over the course of a few days. The addition of antifungal coverage may be necessary since *Candida* species is a possible infectious culprit. NGT decompression may be necessary especially in the setting of gastric distension on imaging, persistent emesis, and concern for bleeding. However, care must be taken as gastric perforation is a concern in this setting. Surgical exploration is indicated in patients who fail optimal medical management, demonstrate signs of clinical deterioration, and peritonitis [2]. According to a systematic review recently published by Watson et al., EG cases reported after the year 2000 were less likely to undergo surgical exploration (62.5% before 2000 versus 22.2% after 2000) with a lower associated mortality overall (59.4% before 2000 versus 33.3% after 2000) [3]. This reduction in mortality has been partially attributed to the lower rate of surgical intervention in the management of EG. Our approach is to utilize surgical exploration selectively and based on clinical deterioration regardless of CT scan findings with the utilization of EGD as an adjunct to help make the decision to operate in unclear cases.

## 4 Conclusion

EG is a rare condition presenting with findings of intramural gas in the stomach wall with associated signs of systemic toxicity. We present three cases of EG with various severities managed conservatively. Early recognition and the initiation of supportive care and antibiotics is key to prevent progression of this potentially fatal condition. The role of surgical intervention should be limited to cases where conservative therapy fails, or signs of peritonitis develop.

## Funding

No source to be stated.

## Ethical approval

The study is exempt from ethical approval in our institution.

## Consent

Written informed consents were obtained for publication of two of the three cases and accompanying images. A copy of the written consents is available for review by the Editor-in-Chief of this journal on request. The third patient could not be reached despite exhaustive attempts and a letter attesting to that has been provided to the journal.

## Registration of research studies

Not applicable.

## Guarantor

Ann Woodward, MD.

## Provenance and peer review

Not commissioned, externally peer-reviewed.

## CRediT authorship contribution statement

**Hassan Nasser:** Writing - review & editing. **Tommy Ivanics:** Writing - original draft. **Shravan Leonard-Murali:** Visualization. **Dania Shakaroun:** Writing - review & editing. **Ann Woodward:** Supervision.

## Declaration of Competing Interest

No conflicts of interest to be declared.
